# Case report: A case of high virulence and multidrug resistant *Klebsiella pneumoniae* liver abscess in Ningxia, China

**DOI:** 10.1097/MD.0000000000036925

**Published:** 2024-01-12

**Authors:** Liangfang Wang, Gang Li, Xiaohui Hou, Lijun Feng, Zhiyun Shi

**Affiliations:** a General Hospital of Ningxia Medical University, Yinchuan, China; b Ningxia Medical University, Yinchuan, China; c Ningxia Key Laboratory of Clinical Pathogenic Microbiology, Yinchuan, China.

**Keywords:** highly virulent multidrug, *Klebsiella pneumoniae*, liver abscess, resistant *Klebsiella pneumoniae*

## Abstract

**Rationale:**

Highly virulent multidrug-resistant *Klebsiella pneumoniae* (KP) is becoming more and more common in clinical practice, especially the rise of carbapenem-resistant KP in clinical practice, resulting in the emergence of KP liver abscess in Ningxia, China. For the prognosis of liver abscess patients, it is particularly important to identify the types of pathogens and identify antibiotics that are sensitive to the pathogens.

**Patient concerns:**

A 73-year-old man from China presents to our hospital with abdominal pain, jaundice and fever. Patients have no obvious cause of abdominal pain, abdominal distension, and abdominal pain is persistent. Abdominal examination showed hepatomegaly, no tenderness 2 cm from the right costal margin, abdominal distension and other general examinations did not have obvious abnormalities. He had no history of hypertension and diabetes, ERCP was performed for cholangiocarcinoma 1 year before the current visit, and no significant complications occurred.

**Diagnoses:**

His initial diagnosis was obstructive cholangitis, and computed tomographic images and liver drainage fluid bacterial culture and genetic polymerase chain reaction tests later determined that the patient had KP liver abscess.

**Interventions:**

Drainage by liver catheter and antibiotic treatment for 7 weeks.

**Outcomes:**

The patient liver abscess is basically gone.

**Lession:**

It is particularly important to optimize the diagnosis of liver abscess pathogens for timely and effective treatment of patients.

## 1. Introduction

*Klebsiella pneumoniae* (KP) liver abscess (KLA) is an emerging syndrome initially described in 1980s in Taiwan. This condition has a high mortality rate and requires prompt and aggressive treatment. Liver abscess has been considered to be an infection caused by a variety of microorganisms. *Escherichia coli* is the main pathogenic microorganism in patients with basic hepatobiliary diseases and gastrointestinal diseases.^[1]^ In recent years, KP has become an important cause of liver abscess in Asian population. The etiology of most liver abscesses is not clear, so these abscesses are characterized by cryptogenesis.^[2]^ Because KP strains may colonize the human gastrointestinal tract, it is most likely that KP translocations through the gastrointestinal tract lead to the formation of liver abscesses.^[3,4]^ In recent years, KP has become an important cause of liver abscess in Asian people. It is also frequently reported in the coastal areas of eastern China that liver abscess caused by highly virulent KP (HVKP) is relatively rare in Ningxia, China. Here, we describe a case of KLA after ERCP due to repeated biliary tract infection in Ningxia, China.

## 2. Case report

**Figure 1.**
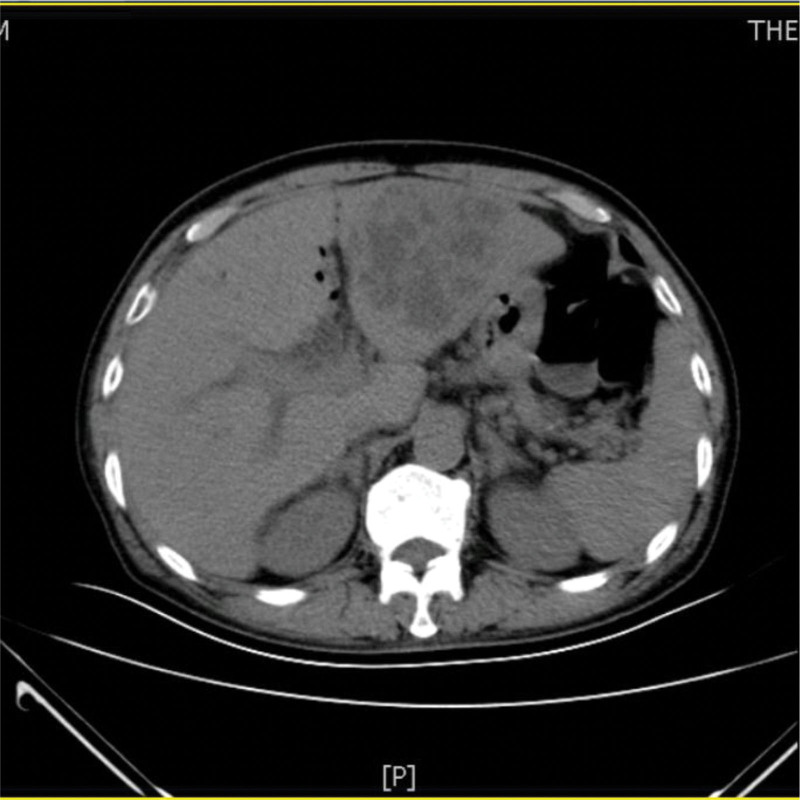
Left lobe liver abscess.

**Figure 2.**
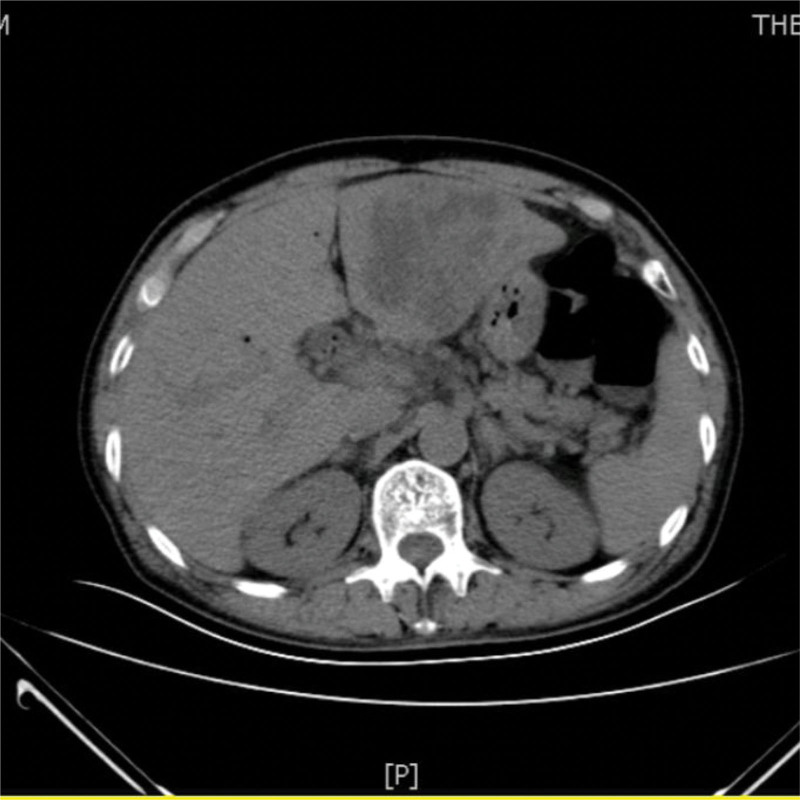
Left lobe liver abscess.

**Figure 3.**
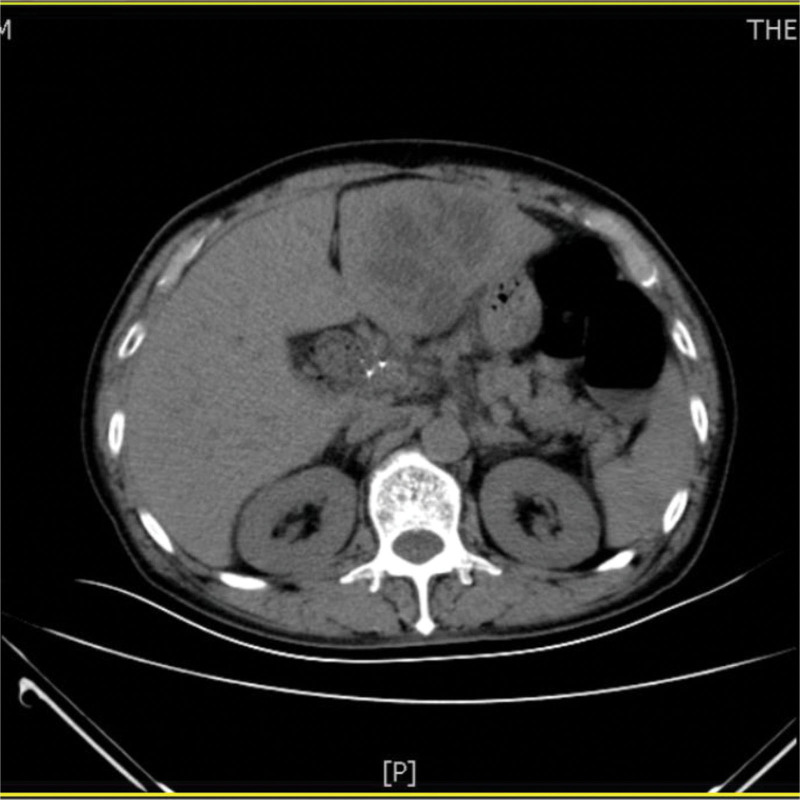
Left lobe liver abscess.

**Figure 4.**
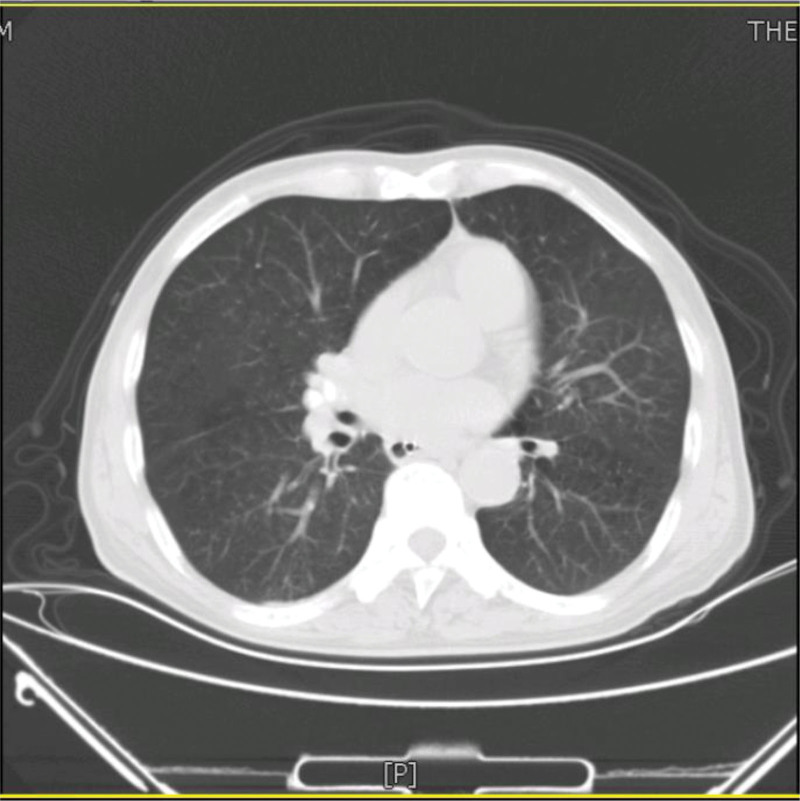
Dispersed inflammation of the right lung.

**Figure 5.**
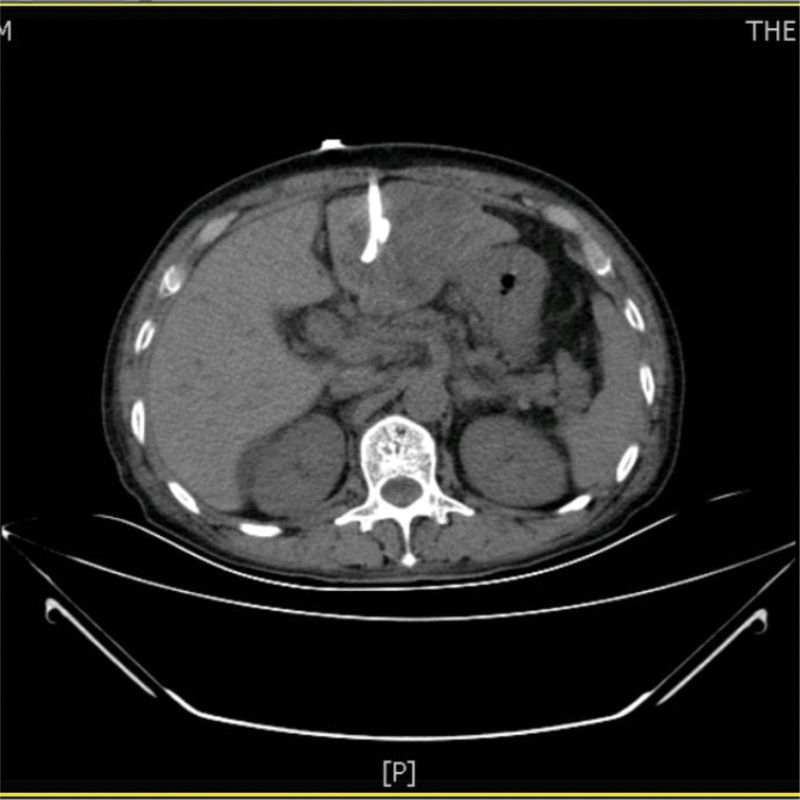
Interventional radiology guided abscess drainage.

**Figure 6.**
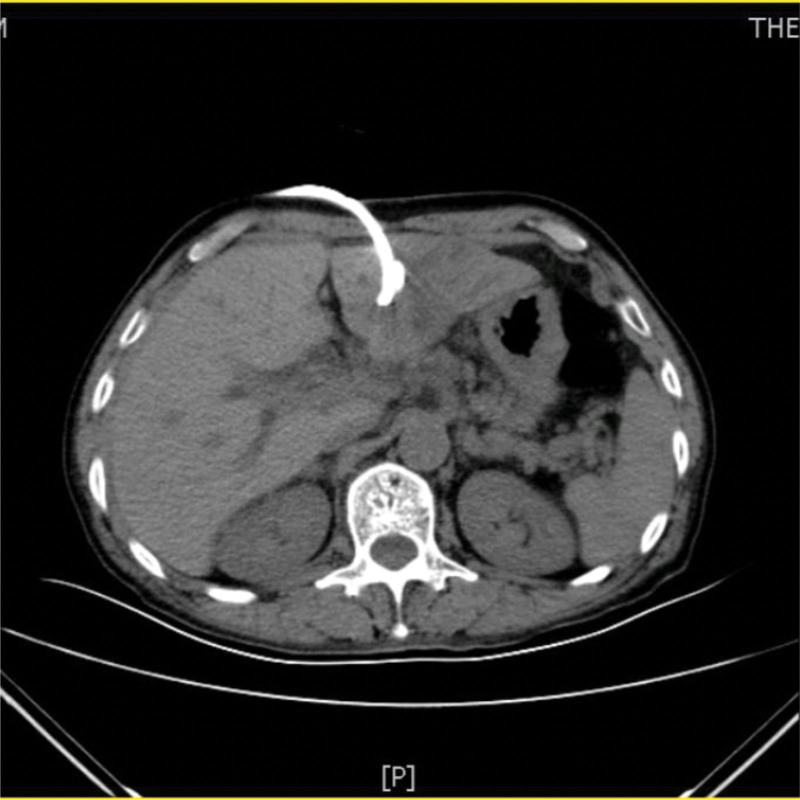
Improvement of liver abscess.

**Figure 7.**
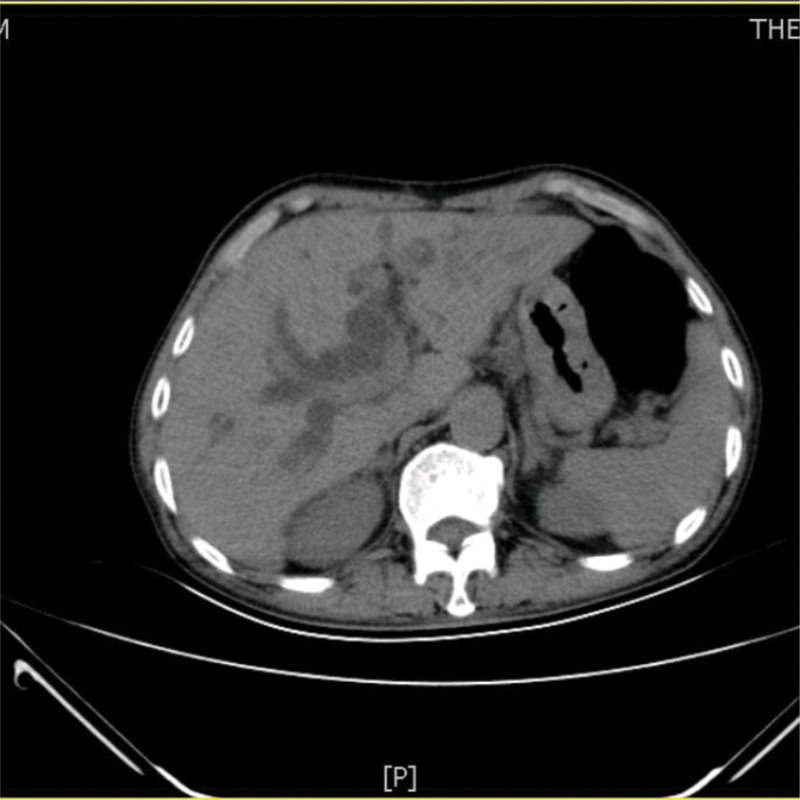
Liver abscess gradually disappears.

**Figure 8.**
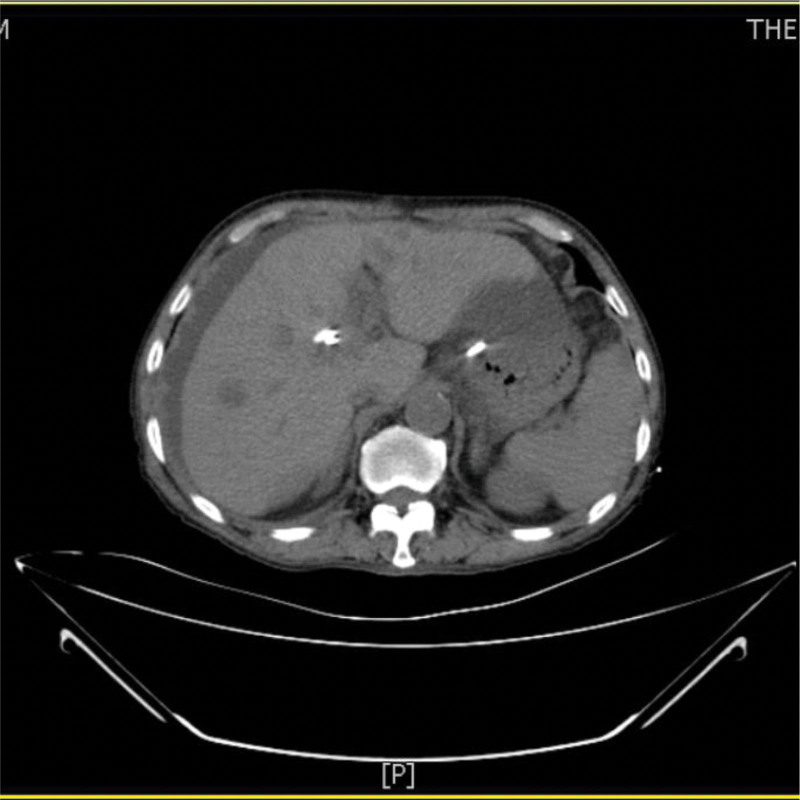
Liver abscess gradually disappears.

**Figure 9.**
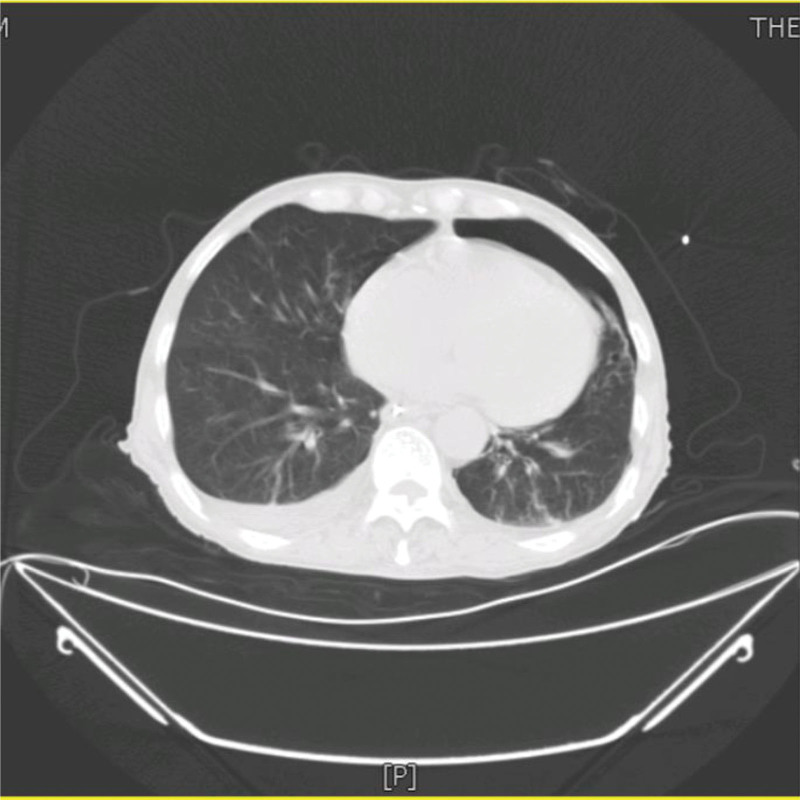
Pleural effusion and pulmonary inflammation.

A 73-year-old man from China visited our hospital on with abdominal pain, jaundice and fever. His initial diagnosis was obstructive cholangitis, the patient was treated with drainage and antibiotics. ERCP was performed for cholangiocarcinoma 1 year before the current visit, and no significant complications occurred. On September 24, 2017, the patient visited our hospital again due to abdominal distension. The patient was treated with diet-deprivation, gastrointestinal decompression, gastric tube drainage, enema and prophylactic use of cephalosporins, he was discharged after his condition improved. Four months later, the patient had no obvious inducement of abdominal pain, abdominal distension, abdominal pain for persistent colic. Abdominal examination showed hepatomegaly 2 cm away from the right costal margin without tenderness, abdominal distention and other general examinations without obvious abnormality. The complete blood count showed a white blood cell count of 6.63 × 10^9^ L, mainly neutrophil, the relative value of neutrophil was 88.1%, the relative value of lymphocyte was 5.1%. Activated partial prothrombin time is 39.6 seconds, the activated partial prothrombin time ratio is 1.45, the fixed quantity of fibrinogen is 6.062 g/L, and the D-D polymer quantity is 7.250. Procalcitonin: 2 ≤ PCT < 10. His urea is 14.23 mmol/L and creatinine 139.2 µmol/L. His fasting blood glucose level was 7.85 mmol/L. Albumin, globulin and white-cell ratio all decreased in varying degrees. Computed tomographic (CT) scan revealed an inhomogeneous low-density lesion in the left lobe of the liver, approximately 7.2 * 6.4 cm in diameter, with poorly defined margins and septa, enhanced posterior wall, and markedly enhanced septa (Figs. 1–3). The patient also had scattered inflammation in the right lung (Fig. 4), we performed interventional radiology-guided abscess drainage (Fig. 5) and performed bacterial culture plus drug sensitivity tests on the drainage and treatment with broad-spectrum antibiotics for 2 weeks. Ten days later, the patient liver abscess improved (Fig. 6) and his mental status improved. KP was positive in culture. The strains were identified as HVKP by clinical definition, virulence phenotype and virulence gene test, and were resistant to extended-spectrum β-lactamases. The patient had a KLA and was treated with long-term antibiotic therapy for 7 weeks. Liver abscesses gradually disappeared after drainage and anti-infective treatment (Figs. 7 and 8). One month later, the patient was reexamined because of fever. CT showed that the liver abscess disappeared, but multiple hepatic cysts, pleural effusion and pulmonary inflammation were found, the imaging findings improved (Fig. 9).

## 3. Genetic analysis

**Figure 10.**
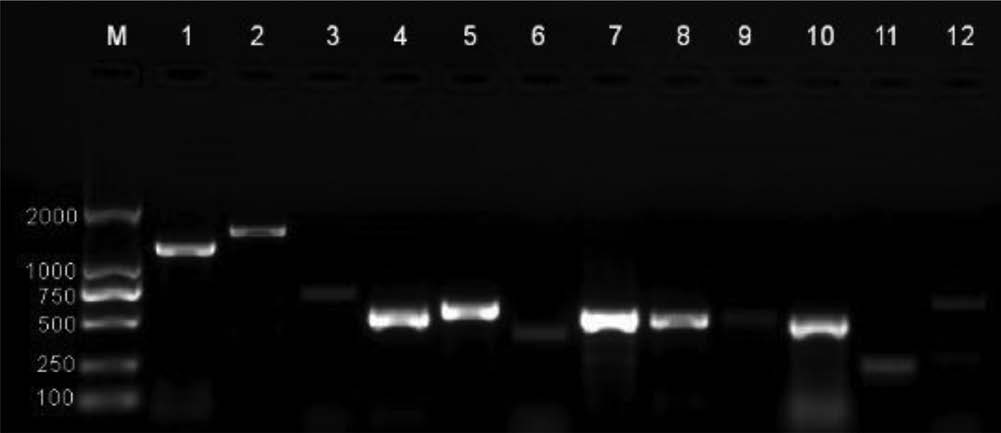
Agarose gel electrophoresis.

Polymerase chain reaction (PCR) assays of the isolates from the case were performed, and revealed the presence of multiple virulence genes, including fim-H, rmpA, Aero, ent-B, alls, mrkD, Kfu, magA, ybtS, iroN. K1 and K57 were detected in its capsule serotype gene. Multilocus sequence typing (MLST) was performed according to the methods of Diancourt et al.^[5]^ The MLST revealed sequence type 23 in the case (Fig. 10). The hypermucoviscous phenotype can easily be confirmed using the string test.^[6]^ In this context, a positive result is defined as bacterial colonies on an agar plate stretching for > 5 mm using the inoculation. The strains isolated from this case also detected ESBLs_TEM_ and ESBLs_SHV_ resistant genes (Fig. 10).

## 4. Discussion

Liver abscess is a potentially life-threatening infection. Common causes of liver abscess include biliary tract disease, gastrointestinal malignancy, and pyelonephritis.^[7]^ It is usually an infection caused by a variety of microorganisms, including *E coli, Streptococcus, Enterobacteriaceae* and anaerobic bacteria, which are well documented in the literature.^[1,8]^ But there is a new trend of primary liver abscess caused by KP.

K1 and K2 were the main serotypes of KP. An alarming incidence of new syndromes caused by KP serotypes K1 and K2 has recently been reported, mainly in Taiwan.^[6,9]^ It is not clear why the Southeast Asian cases dominate, and the Genetic predisposition remains unconfirmed. However, in Southeast Asian individuals, fecal carrier rates were higher for the virulent K1 KP strain ST23, which may account for the higher incidence.^[10]^ Intestinal colonization of virulent strains has been shown to be highly correlated with the occurrence of KLA.^[11]^ This genotype is closely associated with highly aggressive disease in Taiwan and has been reported on every continent, indicating its widespread geographical presence.^[12]^ KLA is now considered to be a new infection and an important cause of endophthalmitis that threatens vision.^[9,13]^

Current studies have shown that capsular serotyping (K1, K2, etc) and locus sequence typing (ST23, ST65, ST86, etc) are usually associated with hypervirulence of KP, but these capsular typing and sequence typing may not have genes that confer hypervirulence, such as K1 and K2 are also expressed in KP. Genetic determinants of hypervirulence are typically present on virulence plasmids and chromosomal mobile genetic elements that serve as biomarkers to distinguish between HVKP and classical KP. With the development of genomics, virulence plasmids (pLVPK, pK2044, pVir-CR-hvK4) and integrated binding elements (ICEkp10) were identified by the HVKP strain. In this study, we performed genetic analysis on KP by PCR and MLST technology, and determined that the patients infected with KP contained fim-H, rmpA, Aero, ent-B, alls, mrkD, Kfu, magA, ybtS, iroN and other virulence genes. The capsular serotype is mainly K1, and the MLST revealed sequence type 23 in the case. The strains isolated from this case also detected ESBLsTEM and ESBLsSHV resistant genes. The analysis of pathogenic bacteria infected by patients through PCR and MLST technology can determine the virulence and drug resistance of pathogenic bacteria, which plays an important role in guiding clinical treatment. With the development of genomics, the whole genome sequencing technology of pathogenic bacteria is becoming more and more widely used, and the application of whole genome sequencing technology can obtain accurate genome information of pathogenic bacteria, and guide clinical treatment through virulence genes and drug resistance genes, which is an area that we need to focus on in the future.

In this case, although the patient was not diagnosed with diabetes, his blood glucose level was poorly controlled and fasting blood glucose was always above normal. In the literature, patients with diabetes are more likely to develop KLA and related septic complications. In addition, regardless of etiology, they developed fever and prolonged hospital stay after treatment.^[14,15]^ Poor glycemic control impairs phagocytosis of K1/K2 KP. Thus, poorly controlled diabetes increases susceptibility to K1/K2 KP infection and associated liver abscess and complicated endophthalmitis.^[16]^ In addition, KLA is strongly associated with colorectal cancer, particularly in the sigmoid and rectum, in East Asian male populations.^[17]^

Many extrahepatic complications of KLA reported in the literature include meningitis, bacteremia with multiple metastatic abscess formation, epidural abscess formation, necrotizing fasciitis, septic arthritis, and septic pulmonary embolism.^[18–21]^ Among them, endophthalmitis threatening vision is often reported.^[22]^ Diabetes mellitus is an important risk factor for endophthalmitis, which indicates a bad visual outcome.^[23]^

At present, the main pathogen of purulent liver abscess in China is KP, and *E coli* has dropped to the second place. In this case, the liver abscess puncture fluid culture supported the etiology of KP, and the diagnosis of KLA was clear, through local medication, CT-guided liver abscess puncture and drainage surgery, and systemic use of antibiotics such as β lactams, fluoroquinolones, carbapenems, etc, achieved good efficacy, but repeated fluctuations in body temperature during treatment also suggested that KP with high virulence was more aggressive, although it was sensitive to most antibiotics, However, it has also been reported that Penemem-resistant KP has a high virulence and a fatality rate of up to 100%, and the combination of virulence and resistance may make it a “superbug” of the future.

In summary, the clinical manifestations of this patient were typical, and early anti-infection and surgical treatment achieved the purpose of improving the prognosis and survival rate of the patient. It can be seen that liver abscess caused by highly virulent and drug-resistant KP has a rapid onset and rapid progression, which requires early diagnosis and early treatment by clinicians to achieve the therapeutic effect. At present, there is no consensus on the definition of KP with high virulence, and more scholars are needed to study together to contribute to clinical work.

## Author contributions

**Conceptualization:** Zhiyun Shi.

**Data curation:** Liangfang Wang.

**Funding acquisition:** Zhiyun Shi.

**Investigation:** Liangfang Wang, Gang Li.

**Validation:** Xiaohui Hou, Lijun Feng.

**Writing – original draft:** Liangfang Wang.

**Writing – review & editing:** Zhiyun Shi.
